# Can a time varying external drive give rise to apparent criticality in neural systems?

**DOI:** 10.1371/journal.pcbi.1006081

**Published:** 2018-05-29

**Authors:** Viola Priesemann, Oren Shriki

**Affiliations:** 1 Max Planck Institute for Dynamics and Self-Organization, Göttingen, Germany; 2 Bernstein Center for Computational Neuroscience Göttingen, Göttingen, Germany; 3 Department of Cognitive and Brain Sciences, Ben-Gurion University of the Negev, Beer-Sheva, Israel; 4 Zlotowski Center for Neuroscience, Ben-Gurion University of the Negev, Beer-Sheva, Israel; Radboud Universiteit Nijmegen, NETHERLANDS

## Abstract

The finding of power law scaling in neural recordings lends support to the hypothesis of critical brain dynamics. However, power laws are not unique to critical systems and can arise from alternative mechanisms. Here, we investigate whether a common time-varying external drive to a set of Poisson units can give rise to neuronal avalanches and exhibit apparent criticality. To this end, we analytically derive the avalanche size and duration distributions, as well as additional measures, first for homogeneous Poisson activity, and then for slowly varying inhomogeneous Poisson activity. We show that homogeneous Poisson activity cannot give rise to power law distributions. Inhomogeneous activity can also not generate perfect power laws, but it can exhibit approximate power laws with cutoffs that are comparable to those typically observed in experiments. The mechanism of generating apparent criticality by time-varying external fields, forces or input may generalize to many other systems like dynamics of swarms, diseases or extinction cascades. Here, we illustrate the analytically derived effects for spike recordings *in vivo* and discuss approaches to distinguish true from apparent criticality. Ultimately, this requires causal interventions, which allow separating internal system properties from externally imposed ones.

## Author summary

The analysis of complex systems in nature introduces several challenges, because typically a number of parameters either remain unobserved or cannot be controlled. In particular, it can be challenging to disentangle the dynamics generated within the system from that imposed by the environment. With this difficulty in mind, we reinvestigate the popular hypothesis that neural dynamics is poised close to a critical point. Criticality is characterized by power-law scaling and has been linked to favorable computational properties of networks. Power-law distributions for “neural avalanches,” i.e., spatio-temporal clusters of neural activity, have been observed in various neural systems and support the criticality hypothesis. Here we show that approximate power laws do not necessarily reflect critical network dynamics but can be imposed externally on non-critical networks, i.e., by driving the network with input of specific statistics. We derive these results analytically and illustrate them both in simulations and using neural recordings. The findings indicate that more caution and additional tests are required for distinguishing between genuine and apparent criticality. Ultimately, this requires causal interventions, not only in neural systems, but in many other complex dynamical systems that are subject to time-varying external forces, such as the dynamics of swarms, diseases or extinction cascades.

## Introduction

In the quest to understand the principles that govern collective neural dynamics, it has been proposed that brains operate at or near criticality [1–5], i.e., a dynamical state that arises at second-order phase transitions and is characterized by scale-invariant activity cascades or avalanches. Criticality is an important candidate state for brain function, because in models criticality optimizes information processing capacities [6–9]. Since the expected power law distributions for avalanches have been found for neural activity on many scales – from spiking activity *in vitro* [10–12] to local field potential, EEG, MEG and BOLD signals in humans [13–18] – these power laws are taken as evidence that the brain does indeed operate at criticality. However, it is known that power laws can also be generated by alternative mechanisms [19]. Most of those mechanisms do not map naturally onto neural networks and are therefore not plausible. However, here we identify a particular mechanism, namely, time-varying changes in the strength of an external drive, as a potential candidate to generate approximate power law scaling in the absence of criticality. Specifically, we investigate the hypothesis that a generic model of neural network dynamics, implemented by an inhomogeneous Poisson process (IPP), can give rise to power law avalanche size and duration distributions.

In the following sections, we outline the conditions under which approximate power law scaling for avalanches arises from IPPs. Specifically, we first derive analytically the duration and size distributions for a *homogeneous* Poisson process (HPP) and show that they follow (approximate) exponential distributions, with rate-dependent decay constants. Subsequently, we derive the known result that superposition, i.e., a weighted summation, of such exponential distributions with different decay constants could, in theory, lead to power laws with any exponent. However, this mechanism does not apply to neural activity, because the weighting function of the rates that is required for a perfect power law cannot be normalized. Hence, this mechanism can generate only approximate power laws with cutoffs. Finally, we show how these approximate power laws can be generated by IPPs and how they resemble avalanche distributions that are typically observed experimentally. Thus, they can, in principle, be mistaken as evidence for criticality.

This paper focuses on the conditions leading to power law distributions from Poisson activity, but power laws form only one marker for criticality. To distinguish apparent criticality from true criticality, it is advisable to extend the criticality analysis beyond power laws. By applying additional measures and by studying the impact of the temporal scale (bin size), many types of IPP can be distinguished from critical processes. In Section 3.3, we also present a number of measures that aid in distinguishing apparent criticality from true criticality, in the hope that this overview will serve as a guide for future rigorous analysis of critical systems. However, it is necessary to bear in mind that because of the correlative nature of any data analysis, a very sophisticated external drive (i.e., very specific IPPs) could perfectly mimic the neural activity of critical systems. Thus, ultimately, the distinction between criticality and apparent criticality can be achieved only by causal interventions that probe the internal system dynamics and disentangle it from the impact of some hidden drive. This idea not only holds for the analysis of critical systems but also points to the fundamental limitation of correlative system analysis in general, which can be overcome only by causal intervention. Nonetheless, even without causal intervention, analyses that go beyond the standard set of avalanche measures can increase the confidence that a particular system is critical.

## Results

**Fig 1:**
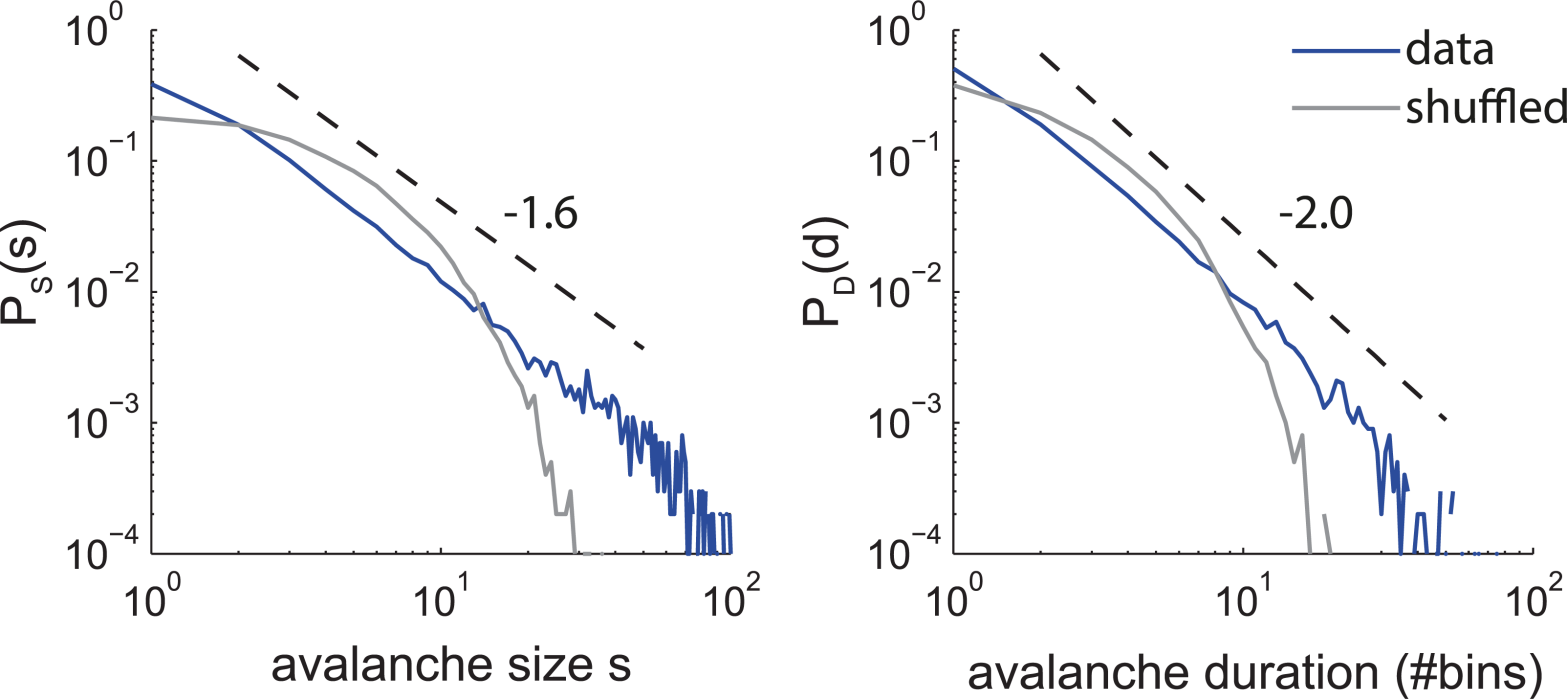
(Color) Avalanche size and duration distributions from an inhomogeneous Poisson process (IPP, blue, see methods), which approximates power laws that resemble those observed in typical experiments. Shuffling the events of the IPP results in a homogeneous Poisson process (HPP, gray). For HPPs, the size and duration distributions are (approximately) exponential rather than following a power law. Dashed lines indicate power laws with exponents -1.6 and -2.0 in the left and right panel, respectively.

We start by asking whether non-critical systems can indeed appear critical. Fig 1 depicts distributions for avalanche sizes and durations that resemble distributions often observed in experiments [4,13]. The distributions exhibit approximate power law scaling with exponents near the theoretical values of -1.5 and -2 for size and duration, respectively. Similar to experimental data, they differ clearly from the avalanche distributions obtained after shuffling the events in the data (Fig 1, gray, thin line). The shuffled data are better fitted by exponential than by power law distributions. Together, these distributions could be taken as evidence for criticality of the underlying systems. However, here the avalanches were actually generated by an IPP. In this example, the process was composed of four equal periods with a different fixed-rate at each period (rates r = {1, 2, 5, 10}/18, i.e., mean rate 1, see Methods). This is a striking example to show that slow and moderate variations in the overall rate of a Poisson process can lead to approximate power laws in the size and duration distributions, which could be mistaken as indicators of criticality. In this paper, we derive the conditions under which IPP can give rise to approximate power laws such as these.

### Results for a homogeneous Poisson process with rate *r*

In this section, we review the avalanche analysis, discuss the impact of the bin size parameter, and then derive analytically the duration and size distributions for an HPP. We also derive or review other measures, including the avalanche shape, the scaling of the shape with duration, the inter-event/avalanche distributions, the spike-count ratio or branching parameter *Q*, the power spectrum and the Fano factor.

#### Definition of neuronal avalanches using temporal binning

Avalanches are defined as cascades of events that originate from a single seed event [20]. For neural recordings, these events are either spikes or binary events obtained from thresholding continuous signals, such as LFP or EEG signals. The events from all recording channels are combined into a single time series of events, *A*(*t*). To extract neuronal avalanches, this time series is partitioned into temporal bins Δ*t*. An avalanche is then defined as a sequence of consecutive non-empty bins. The duration of an avalanche is the number of bins, and its size is the total number of events. Subsequent avalanches are separated by at least one empty bin. These empty time bins, or pauses, between any two avalanches, are characteristic of critical systems [4,20–22]. However, technically, avalanche analysis can be applied to any time series that shows pauses, such as the Poisson processes we are analyzing here. Importantly, the choice of the bin size can impact the avalanche distributions. Thus, for any data or model analysis, taking this bin size dependence into account adds valuable information about the system.

*Effect of the bin size and rate*. In all derivations, the rate *r* is typically varied, whereas the bin size (Δ*t*) is fixed. Here, without loss of generality, Δ*t* = 1 ms, and this bin size often equals one average *IEI*, namely, Δ*t* = 1/*r* = 〈*IEI*〉. It is sufficient to derive only the dependence of the different quantities on *r*, because the HPP depends only on the product *r* Δ*t*. As a consequence, changing Δ*t* to Δ*t*′ is strictly equivalent to changing the rate of the HPP from *r* to *r*′ = *r* ∙ Δ*t*′/Δ*t*. Thus, exploring rate dependences is exchangeable with exploring bin size dependences.

*Contribution of each neuron*. How does each single neuron contribute to the population activity *A*(*t*)? In our generic network model, we assume that each neuron follows the same rate envelope or drive *r*(*t*). For the HPP, *r*(*t*) *= r* is constant. Each neuron *i* can spike with its own average rate *r*_*i*_. Thus, although rates can differ among neurons, the sum of the rates over all *N* recorded neurons must equal the rate of the process, $\sum_{i=1}^{N}r_{i}=r$. For the resulting *A*(*t*), it is equivalent to either double the number of neurons or to double the rate of each neuron. Importantly, as in IPPs, all neurons follow the same drive *r*(*t*). This common drive introduces correlations between the neurons' firing, and these correlations contribute to the long-tailed avalanche distributions.

### Analytical derivation of avalanche duration and size distributions

For an HPP, it is commonly assumed that the avalanche measures are exponential and not power law distributed. We show analytically that the duration distribution, *P*_*D*_(*d*), is indeed exponential, but the expression for the size distribution, *P*_S_(*s*), deviates from the exponential assumption. In the main text we provide the results together with the outline of the derivation, and the full analytical derivations are detailed in the Methods section.

**Fig 2:**
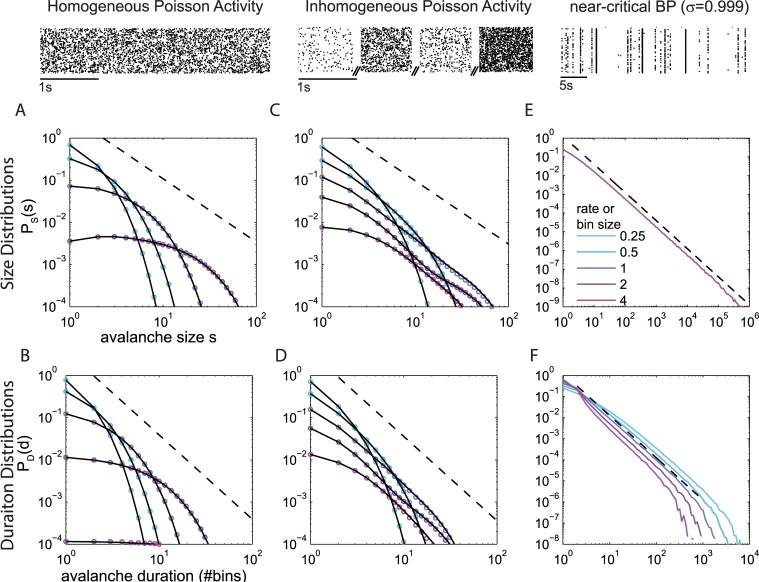
(Color) Avalanche size and duration distributions for three example processes, as exemplified in the raster plots above, all with the same mean rate: **A&B**. homogeneous Poisson process, **C&D**. inhomogeneous Poisson process, **E&F**. critical branching process (BP). Different colors represent different bin sizes, Δ*t*, at *r* = 1 (or equivalently different rates *r* at Δ*t* = 1). Colored lines or dots are numerical results; black lines are analytical results. **A-D.** For both the homogeneous and the inhomogeneous Poisson processes, an increase in Δ*t* (or *r*) makes the size distribution *P*_S_(*s*) and the duration distribution *P*_*D*_(*d*) flatter. **E&F.** For the critical system, a change in Δ*t* (or *r*) hardly changes *P*_S_(*s*), which shows a power law with exponent -1.5 (dashed). The slope of *P*_*D*_*(d*) changes systematically, because *d* is in units of bins. In units of time steps, *P*_*D*_*(d*) would also change very little and show the exponent -2 (dashed).

*Avalanche duration distribution P*_*D*_(*d*). Consider an HPP with rate *r* and bin size Δt = 1 time step. The avalanche duration *d* is defined as the number of non-empty bins in a sequence. The probability of a bin being empty is *p*_0_ = e^−*r*^, and the probability of a non-empty bin is thus *p* = 1 − *p*_0_ = 1 − e^−*r*^. Because the events in different time bins are independent, the probability of obtaining a sequence of *d* non-empty bins between two empty bins is proportional to $p^{d}p_{0}^{2}$. This gives P_D_(d) ~ (1 − *e*^−*r*^)^*d*^ = e^−*μ*(r)d^, where *μ*(*r*) = −ln(1 − *e*^−*r*^) is the rate-dependent decay constant of the exponential. Thus, the avalanche durations are exponentially distributed, and the distributions become flatter as *r* increases (Fig 2B). The normalized distribution is given by (see Methods):

$$P_{D}(d)=\frac{1}{Z_{D}}p_{0}^{2}p^{d}={e^{-rd}(e^{r}-1)}^{d-1}$$ (1)

The above results hold for any rate (or equivalently for any bin size). For the widely used bin size of one “average inter-event interval,” Δ*t* = 〈*IEI*〉 = 1/*r*, the duration distribution is independent of the rate *r* and simplifies to:

$$P_{D}(d\;|\;\Delta t=1/r)={\frac{1}{e-1}(\frac{e-1}{e})}^{d}$$ (2)

*Avalanche size distribution P*_*S*_(*s*). The derivation of the avalanche size distribution *P*_S_(*s*) is more intricate than the derivation of *P*_*D*_(*d*) (see Methods for full details). The first step involves obtaining an expression for the conditional size distribution, *P*_*S*_(*s*|*d*). This requires knowing the probability of having *A* = *a* events in a bin, which is given by the Poisson distribution, $P_{A\geq 0}(a)=\frac{r^{a}e^{-r}}{a!}$. However, within an avalanche, all bins have *a* ≥ 1 events, and therefore the probability must be renormalized, yielding:

$$P_{A\geq 1}(a)=\frac{P_{A\geq 0}(a)}{1-p_{o}}=\frac{r^{a}}{(e^{r}-1)a!}$$ (3)

The size of each avalanche is then the sum of the events from all the bins that constitute it, namely, the sum of *d* independent random variables *A*. The conditional size distribution *P*_*S*_(*s*|*d*) can be derived from the corresponding probability-generating function (see Methods). The resulting expression involves Stirling numbers of the second kind, $\left\{\begin{array}{c} s\\ d \end{array}\right\}$, which represent the number of ways to distribute *s* events into *d* bins such that none of the bins is empty (the number of surjections from *s* to *d*):

$$P_{S}(s|d)=\frac{r^{s}d!\{\begin{array}{c} s\\ d \end{array}\}}{s!{\left(e^{r}-1\right)}^{d}}=\frac{r^{s}[\sum_{i=0}^{d}{\left(-1\right)}^{i}(\begin{array}{c} d\\ i \end{array}){\left(d-i\right)}^{s}]}{s!{\left(e^{r}-1\right)}^{d}}$$ (4)

In the second step, *P*_*S*_(*s*|*d*) is combined with *P*_*D*_(*d*) to yield the size distribution:

$$P_{S}(s)=\sum_{d=1}^{s}P_{S}(s|d)P_{D}(d)$$

$$=\sum_{d=1}^{s}\frac{r^{s}[\sum_{i=0}^{d}{\left(-1\right)}^{i}(\begin{array}{c} d\\ i \end{array}){\left(d-i\right)}^{s}]{\left(e^{r}-1\right)}^{d-1}}{s!{\left(e^{r}-1\right)}^{d}e^{rd}}=\frac{r^{s}}{s!}\frac{1}{\left(e^{r}-1\right)}\sum_{d=1}^{s}e^{-rd}\sum_{i=0}^{d}{\left(-1\right)}^{i}(\begin{array}{c} d\\ i \end{array}){\left(d-i\right)}^{s}$$ (5)

This distribution is not exponential and does not resemble a power law (Fig 2A). Note that it does also not necessarily decrease monotonically with *s*. In fact, for large enough rates, *r*>2, *P*_*S*_(*s*) shows a global maximum at *s*>1. However, the tail of the distribution approximates an exponential (see Methods). More precisely, for large *s* the distribution can be approximated by:

$$P_{S}(s)\approx \lambda e^{-\lambda s},$$ (6)

where *λ* is a function of *r*

$$\lambda (r,s_{c})=\lim_{s\to \infty }-\log\;\frac{P_{S}(s+1)}{P_{S}(s)}=-\log\;r-1+B(s_{c})$$ (7)

*B*(*s*_*c*_) accounts for the slow change of *λ* with *s* and is evaluated for a representative *s*_*c*_ (see Methods). Thus, in contrast to the duration distribution, the size distribution is not exponential and is not necessarily monotonic.

### Additional avalanche measures

**Fig 3:**
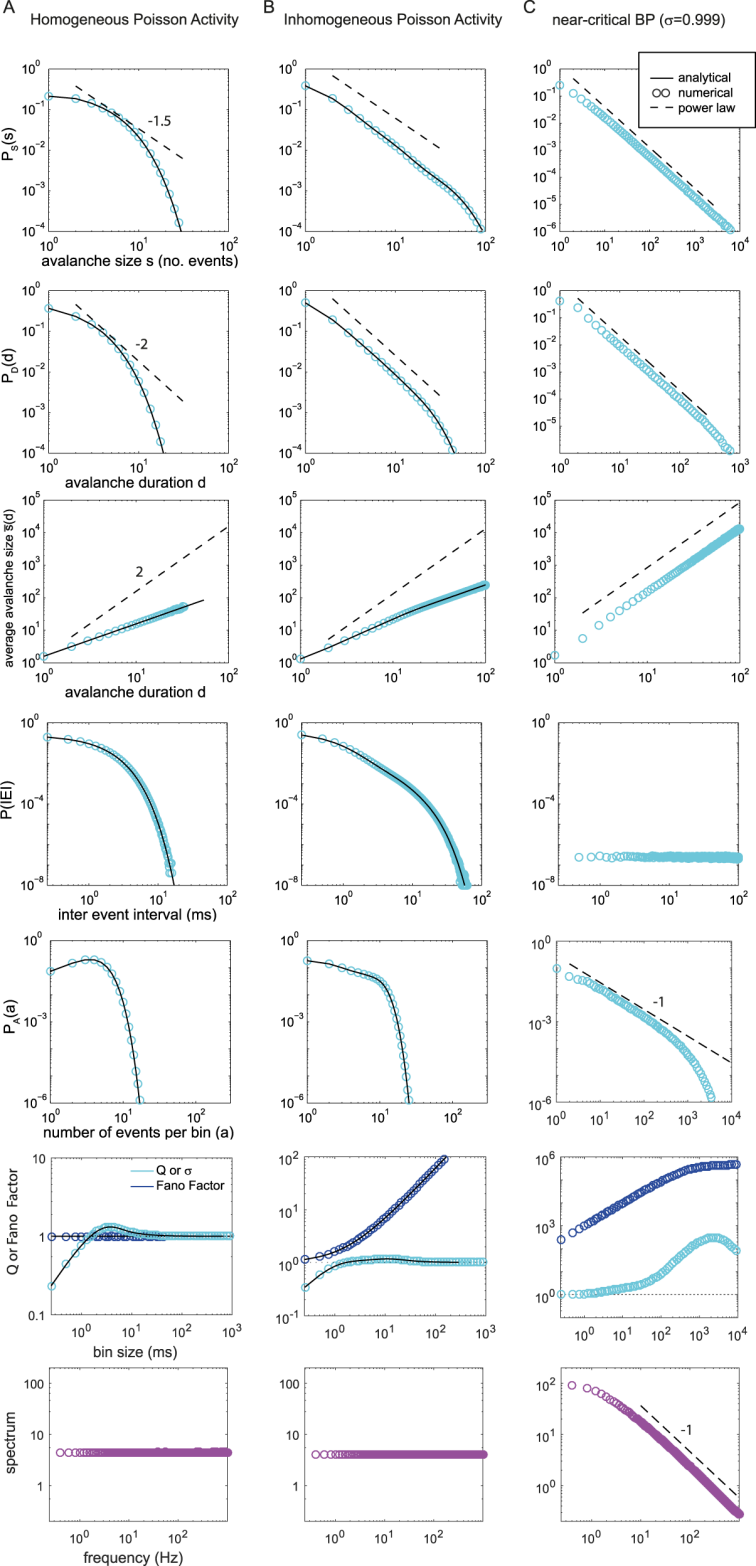
(Color) Avalanche size and duration distributions and additional time-series measures for: **A.** a homogeneous Poisson process (HPP), **B.** an inhomogeneous Poisson process (IPP, same as Fig 1), and **C.** a near-critical branching process (BP) with branching parameter σ = 0.999 = *σ*_*c*_ − 10^−3^. Circles represent numerical results; black lines represent analytical results; and dashed line are reference power laws. The mean rate of all processes is *r* = 1, and the bin size (if relevant) is Δ*t* = 1. The top row shows representative examples of raster plots for each process. While HPPs do not follow power laws, the avalanche size distribution of the example IPP does approximate a power law with cutoff, comparable to distributions obtained in experiments and in simulations of critical branching processes.

In this section we derive or review additional common time series measures for the HPP. All results are shown in Fig 3A.

*Avalanches shape*. In critical systems, the avalanche shape is expected to be "universal," i.e., the characteristic shape *F*^*u*^(*t*/*d*) of the avalanche scales with the duration *d* of the avalanche *F*(*t*,*d*) = *F*^*u*^(*t*/*d*)*d*^*ν*^ [23,24]. This relationship implies that the average avalanche size $\bar{s}$ also scales with *d*. For homogeneous Poisson processes, the shape is flat, because the expected number of events per bin of size 1 is simply *r*. The expected size $\bar{s}$ for a given duration is thus:

$$\bar{s}(d)=d\cdot r$$ (8)

Thus, $\bar{s}(d)$ follows a trivial power law with slope *ν* = 1, or, more simply, $\bar{s}$ is proportional to *d* (Fig 3A). For certain critical systems, specific relations between the exponents of *P*_S_(*s*), *P*_D_(*d*), and $\bar{s}(d)$ have been predicted [23,24]. However, for HPPs neither *P*_S_(*s*) nor *P*_D_(*d*) follows a power law, and thus the scaling relationships are not applicable.

*Inter-event and inter-avalanche-interval distributions*. The inter-avalanche-interval (*IAI*) distribution is closely related to the *IEI* distribution of *A*(*t*), that is the *IEI* is calculated from taking all events together. More precisely, the *IAI* distribution is a left-truncated version of the *IEI* distribution, where the truncation is determined by the bin size. In other words, all *IEI*s that are smaller than Δ*t* do not contribute to *P*(*IAI*), whereas for all *IEI* or *IAI* > 2Δ*t*, the counts for *IEI* and *IAI* are exactly equal. As *P*(*IEI*) is the more general distribution, we report only *P*(*IEI*) here. Analytically, *P*(*IEI*) is the inter-event distribution of a Poisson process

$$P(IEI)={re}^{-r\;IEI}$$ (9)

and follows an exponential (Fig 3A). Note that even under very high rates (*r* ≫ 1), there is still a non-zero probability of obtaining empty bins. This allows parsing the process into avalanches.

*Fano factor F*. The Fano factor is the mean-normalized variance of a process and for Poisson processes *F*=1, independently of the bin size (Fig 3A).

*Event or spike count ratio Q*. The spike count ratio (or branching parameter), *Q*, is defined as the expected value of activity in one bin divided by the activity in the previous bin, $Q(\Delta t)=\langle \frac{A(t+1|\Delta t)}{A(t|\Delta t)}\rangle$, and the expectation is taken over all bin pairs with *A*(*t*|Δ*t*) ≥ 1 [4]. For HPPs, *Q* can be derived analytically. *Q* changes with Δ*t*, and, as before, the dependence on Δ*t* is equal to that on *r*, i.e., *Q*(Δ*t* = *z*|*r* = 1) = *Q*(*r* = *z*|Δ*t* = 1). The analytical expression for *Q* for Poisson processes is derived in the Methods. It yields:

$$Q(\Delta t)=\frac{\Delta t\;(\ln\left(\Delta t\right)+\gamma -Ei(\Delta t))}{1-e^{\Delta t}}$$ (10)

where *Ei*(Δ*t*) is the exponential integral, and *γ* is the Euler-Mascheroni constant (*γ* ≈ 0.577) [25]. The spike count ratio *Q*(Δ*t*) increases for small Δ*t*, equals unity for Δ*t* ≈ 1.5 ∙ 〈*IEI*〉, assumes a maximum at Δ*t* ≈ 3.75 ∙ 〈*IEI*〉, and finally approaches unity from above for Δ*t* → ∞ (Fig 3A).

*Fourier spectrum*. Finally, the Fourier spectrum of a Poisson process is known to be flat (Fig 3A, bottom panel).

### Weighted superposition of exponential distributions can yield power laws with a cut-off but not perfect power laws

As derived above, the durations and size distributions of HPPs are (approximately) exponential. The decay constant of the exponentials depends on the rate of the process, *r*. This dependence is the key to obtaining approximate power law distributions from IPPs, via superimposing multiple exponential distributions, which are each generated by periods of activity with different rates. Mathematically, it is known that specific superpositions (i.e., weighted sums) of exponential functions lead to power laws. In this section, we review the general conditions under which such a superposition can lead to a power law with a given exponent. We then translate these conditions to neural activity with a time varying rate (IPP) and show that a perfect power law cannot be obtained. However, superposition of a few exponentials can result in approximate power law distributions, spanning a few orders of magnitude.

To obtain a perfect power law *P*(*x*) ~ *x*^−*α*^ from the superposition of exponentials, the weighting function *w*(*λ*) for each decay rate *λ* must fulfill the following condition:

$$P(x)=\int_{\lambda_{1}}^{\lambda_{2}}d\lambda \;w(\lambda )e^{-\lambda x}∼x^{-\alpha }$$ (11)

Note that here, for the sake of clarity, generic exponential functions *e*^−*λx*^ are first used; later we replace them with the full avalanche duration distributions of HPPs. To obtain a power law without a cutoff, the bounds of the integral have to extend over the entire interval *λ*_1_ = 0 to *λ*_2_ → ∞. Otherwise, the range of the power law distribution is limited on the right or left, respectively. The weighting function that results in a power law is a power law in itself: *w*(*λ*)~*λ*^*α*−1^ (see Methods).

$$P(x)=\frac{1}{Z_{p}}\int_{0}^{\infty }d\lambda \;\lambda^{\alpha -1}e^{-\lambda x}=\frac{\Gamma (\alpha )}{Z_{p}}x^{-\alpha }∼x^{-\alpha }$$ (12)

Γ is the gamma function, *Z*_*p*_ is the normalization, and *α* > 1 to allow normalization of the power law. However, *w*(*λ*)~*λ*^*α*−1^ cannot be normalized for *α* ≥ 0, i.e., the probabilities *w*(*λ*) with which each exponential *e*^−*λx*^ would contribute to the power law are undefined. As a consequence, real-world systems cannot generate a perfect power law from addition (superposition) of exponentials. However, the weights can be normalized by choosing a reduced integration range [*λ*_1_,*λ*_2_] at the cost of obtaining only an approximate power law with cutoffs. This approach is used below to study avalanche distributions generated by an IPP. To achieve this goal, we need first to translate the general relation for *P*(*x*) above to the specific cases of the duration distribution *P*_*D*_(*d*); in particular, we need to derive the specific weight function *w*(*r*)– instead of the generic function *w*(*λ*) – that gives rise to a power law for *P*_*D*_(*d*)~*d*^−*β*^. The density or weighting function *w*(*r*) denotes the fraction of time that an IPP has to assume each rate *r* (and hence sample from the respective exponential distribution), so that a power law is obtained across the full IPP. We assume that the IPP rate changes far more slowly than the typical duration of an avalanche. We can thus assume that an IPP takes a fixed rate *r* for some time window. During each time window, the duration distribution is *P*_*D*_(*d*|*r*), as derived above for fixed rates (HPP, see Eqs (1) and (2)). The resulting *P*_*D*_(*d*) of the IPP can be written as:

$$P_{D}(d)=\frac{\int_{0}^{\infty }dr\;w(r)\rho (r)P_{D}(d|r)}{Z_{D}}∼d^{-\beta }$$ (13)

where *Z*_*D*_ is the appropriate normalization, and *ρ*(*r*) is the rate at which avalanches occur given a Poisson rate *r*. This equation holds, in analogy to the argument above, if all the factors in front of the exponential in *P*_*D*_(*d*|*r*) are proportional to *r*^*β*−1^. This condition yields the general expression for *w*(*r*) (see Methods):

$$w(r)=\frac{{\left(-\log\left(1-e^{-r}\right)\right)}^{\beta -1}}{{(1-e^{-r})e}^{-r}}$$ (14)

To obtain, for example, *P*_*D*_(*d*) ~ *d*^−2^, which is characteristic for critical branching processes, the weighting function is:

$$w(r|\beta =2)=\frac{-\log\left(1-e^{-r}\right)}{{(1-e^{-r})e}^{-r}}$$ (15)

**Fig 4:**
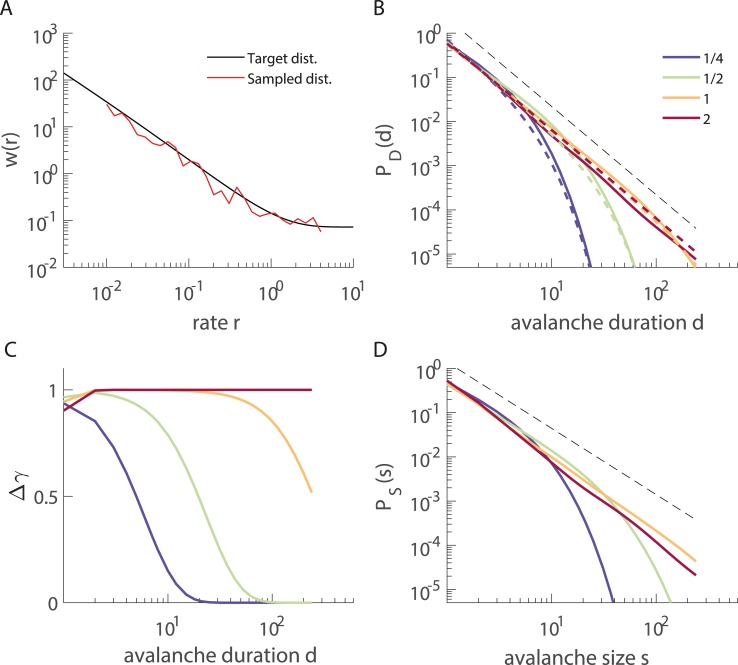
(Color) Superposition of exponential distributions that arise from windows with a fixed Poisson firing rate can combine to power law distributions with a cutoff. **A.** The weighting function *w(r)* that leads to a power law with an exponent of -2 in the duration distribution; the analytical result is shown as a dashed black line, and a specific stochastic realization that we used for panels B-D is shown as a non-broken red line. **B.** Avalanche duration distributions *P*_*D*_(*d*) arising from the weighting function in A, using either the red weighting function (solid line) or the analytical expression with the same integration limits, i.e., 0.01 to 5 (dashed lines). Colors indicate different bin sizes Δt (in units of 1/r), and the dashed black line is a reference power law with exponent -2. **C.** Effect of bin size on the cutoff. The different functions depict the theoretical cutoff function Δ*γ* imposed on the target duration distribution *P*_*D*_(*d*) ~ *d*^−2^ for different bin sizes (see text for details; same color code as in B). **D**. Avalanche size distributions *P*_*S*_(*s*) from the same red weighting function as in A also show an approximate power law with an exponent characteristic of branching processes (exponent -1.5 is indicated by the dashed black line).

This function is approximately 1/*r* for *r* ≪ 1 and approximately constant for *r*>1 (Fig 4A, black dashed line). Importantly, this implies that for low rates the number of events that each rate contributes is invariant: *w*(*r*)*r* ~ 1/*r* ∙ *r* = *const*., whereas for large rates, each rate contributes an equal fraction of time (*w*(*r*) = *const*.), and hence larger rates contribute more events (~ *r*). However, it immediately becomes clear that this weighting function cannot be normalized over the full range of rates from zero to infinity. Nonetheless, *w*(*r*) can be normalized if a limited integration range [*r*_1_,*r*_2_] is chosen, albeit at the cost of introducing a right and left cutoff to the power law of *P*_*D*_(*d*), respectively. For the numerical illustration in Fig 4, we chose the range [*r*_1_ = 0.01, *r*_2_ = 5] and sampled 300 values from *w*(*r*) in this range. For the analytical results, the functional form of the cutoffs can be obtained as follows (see Methods):

$$P_{D}(d)∼d^{-\beta }(\gamma (\beta ,-d\;\log\left(1-e^{-r_{1}}\right))-\gamma (\beta ,-d\;\log\left(1-e^{-r_{2}}\right)))≔d^{-\beta }\cdot \Delta \gamma (\beta ,r_{1},r_{2})$$ (16)

where *γ*(⋅,⋅) is the lower incomplete gamma function. The terms *γ*(⋅,⋅) generate smooth cutoffs on both sides of the power law *d*^−*β*^ by “windowing” it. The windowing function Δ*γ* = Δ*γ*(*β* = 2, *r*_1_ = 0.01, *r*_2_ = 5) is depicted in Fig 4C for different Δ*t* (or *r*). With increasing bin size (or, equally, with increasing rate) it moves to larger avalanche durations *d* (i.e., to the right). Likewise, the cutoffs of the resulting *P*_*D*_(*d*) move from left to right (Fig 4B). The right cutoff is thus prominent at small bin sizes (Δ*t* < 1), whereas the left cutoff sets in at large bin sizes (Δ*t* > 1). For Δ*t* = 1, this example IPP shows a power law that extends over more than two orders of magnitude.

In branching processes, the characteristic exponent for the duration distribution is -2, whereas for the size distributions it is -1.5. Interestingly, we obtained the same pair of exponents for IPPs by naively applying to the size distributions *P*_*S*_(*s*|*r*) the exact weight function *w*(*r*|*β* = 2) that we had derived for *P*_*D*_(*d*). Thus, by construction, for an IPP that gives rise to *P*_*D*_(*d*) ≈ *d*^−2^/*Z*_*D*_ (with cutoff), the corresponding size distribution shows a power law with *P*_*S*_(*s*) ≈ *s*^−1.5^/*Z*_*S*_ when applying a bin size of Δ*t* = 1 (Fig 4D). Avalanches extracted from this IPP can thus easily be taken as evidence for criticality. In summary, Poisson neurons with slowly changing finite rates can give rise to approximate power laws with the characteristic exponents -1.5 and -2 for the sizes and durations, respectively, *if the different rates occur with probability w(r*|*β* = 2) *as derived above*. In practice, the generation of a power law from superimposed exponentials can be realized only with a cutoff and requires the weighted contribution of each exponential according to *w*(*r*|*β*).

### Non-stationary Poisson processes can give rise to approximate power law distributions for avalanches, but typically differ from critical processes in other measures

As shown above, IPPs can give rise to approximate power laws with a cutoff if their rates change slowly and if they are distributed according to *w*(*r*|*β*) on an interval [*r*_1_,*r*_2_]. In this section, we show that the rate distribution does not have to be exactly *w*(*r*|*β*) to generate distributions that resemble those obtained from experimental results. However, IPPs and truly critical processes typically differ in other measures. This differentiation allows us to distinguish apparent critical systems from truly critical systems, as described below.

Consider an IPP that assumes one of four equiprobable rates {*r*_1_,*r*_2_,*r*_3_,*r*_4_} = {0.1,0.2,0.5,1}/*Z*. The normalization *Z* = 5/9 assures 〈*r*〉 = 1, without loss of generality. Each rate is maintained for a long time window compared to the typical avalanche duration—here for 250,000 time steps (≈ 4 min, assuming a sampling rate of 1 kHz). While each interval separately shows (approximately) exponential avalanche distributions, combining all epochs results in an approximate power law with a cutoff at around *s* = 80, thus covering almost two orders of magnitude for *P*_*S*_(*s*) and *P*_*D*_(*d*) and also for *P*(*IEI*), the inter-event interval distribution (Fig 3B, same parameters as Fig 1). Thus, avalanche distributions from this simple non-stationary Poisson process could easily be taken as evidence for criticality, especially since the exponents match those of a critical branching process (-1.5 and -2 for the size and rate distribution, respectively, Fig 3B).

Measures other than avalanche distributions, however, show clear differences between this inhomogeneous Poisson activity (IPP) and a critical branching process (compare Fig 3B and 3C, respectively), as follows: (i)The relationship between mean avalanche size and duration, $\bar{s}(d)$, exhibits an almost perfect power law for the IPP, but not with the exponent of -2 that is expected from the exponents of the size and duration distributions [23,24]. Instead it shows the trivial exponent of unity, i.e., $\bar{s}(d)$ ~ *d*. (ii) The inter-event interval distribution *P*(*IEI*) is flat for the branching process but constitutes a sum of exponentials, approximating a power law, for the IPP. (iii) The density of events *P*_*A*_(*a*) approximates a power law for the branching process but not for the IPP. (iv) The estimated branching ratio or spike count ratio *Q*(Δ*t*) shows a pronounced maximum for the branching process (*max*_Δ*t*_ *Q*(Δ*t*) ≈ 500), whereas for the IPP *Q*(Δ*t*) is close to unity for all Δ*t* ≥ 1. (v) The Fano factor takes much higher values around Δ*t* = 1 in the branching process than in the IPP (note the different y-axis ranges). (vi) The Fourier spectrum of the population activity *A*(*t*) shows a power law spanning more than two orders of magnitude for the branching process, whereas it is flat for the IPP. (vii) Finally, *P*_*S*_(*s*) and *P*_*D*_(*d*) for the IPP change markedly with Δ*t*, as predicted analytically, whereas for the branching process they are almost invariant against moderate changes in Δ*t* (Fig 2C–2F). This is because the critical branching process, despite having exactly the same average rate as the IPP, shows a moderate separation of time scales (see Discussion).

## Discussion

We have shown that it is not possible to generate a perfect power law for avalanches with an IPP, whereas approximate power laws, extending over several orders of magnitude before cutoff, can be generated by assuming that the rates vary over time across only one or two orders of magnitude. Our findings thus indicate that power law distributions for avalanches may also appear in non-critical systems, given a specific time-varying external drive. For many types of input, an analysis that extends beyond avalanches alone can rule out or provide evidence for the criticality hypothesis. However, for certain types of input (in particular *r(t)* of the IPP mimicking exactly the *A(t)* generated by a true critical system), "passive" data analysis, from avalanche size through Fourier spectrum to approaches from equilibrium thermodynamics [26], cannot distinguish between them.

The distinction between a critical-like driven system and a truly critical system ultimately requires manipulation, i.e., the use of “active” causal interventions. Application, for example, of small, controlled perturbations can separate the intrinsic network properties from those imposed by the external input. In critical systems, these perturbations cause avalanches that should follow the predicted power-law distributions. Alternatively, manipulations could directly target the control parameter of the system and assess its impact on the correlation length, susceptibility and specific heat [8,27,28]. Thereby one can establish a second-order phase transition. While such manipulations may be feasible in models, not all experimental preparations allow for well-controlled manipulations, and alternatively manipulating a maximum entropy model fitted to the data may yield spurious results [29]. If direct manipulations cannot be applied, data analyses should make use of the diversity of measures available, including investigating the effects of different temporal bin sizes. Such combined analyses can distinguish between many types of rate-varying drives and truly critical systems. However, again, analyses without manipulations are not sufficient to distinguish between a drive that perfectly mimics the *1/f* envelope expected for critical systems and *1/f* dynamics generated because of criticality within a network. At the core of these considerations is the fundamental issue of correlative versus causal studies of the underlying system. In general, correlative approaches can be "fooled," and thus the more rigorous, causal analysis is advisable.

We have discussed here the superposition of exponentials as a potential alternative mechanism to criticality that may underlie power law generation. A number of other alternative mechanisms have been proposed, four of them compiled by Newman [19], namely, a combination of exponentials, inverses of quantities, random walks, and the Yule process. There are basically two reasons why it is not possible for these alternative models to explain the power laws observed for neuronal avalanches: either the experimentally observed distributions do not agree with the model functions (e.g., the Yule process shows a power law tail, whereas neuronal avalanches show cutoffs; random walks show an exponent of -2, whereas for avalanche sizes it is typically -1.5), or it is not clear how the generating mechanism would map onto neural networks (all four examples). In contrast, the branching process offers an elegant mechanistic approximation of spike propagation on a network and exhibits the same avalanche distributions as those observed in data [4,30,31].

Schwab et al. [32] and Aitchison et al. [33] have shown that power laws for pattern frequency, i.e., Zipf's law, can emerge from a random external input or field. Their studies are similar to ours in that they used a varying external input, in effect, potentially also leading to a superposition of exponentials. However, avalanches – in contrast to Zipf patterns – are temporally extended, and thus the random external field is not sufficient to generate power law avalanche distributions. The spatio-temporal characteristics of avalanches require a *temporally correlated* external field. The effects of such a temporally correlated external field have been studied by Touboule & Destexhe [34]. They, in analogy to our study, applied a time-varying external field *r*(*t*) to all Poisson neurons. They chose one specific *r*(*t*), namely, an Ornstein–Uhlenbeck (OU) process, which they realized with a long correlation time *τ* compared to the bin size Δ*t* of the avalanche analysis. (They chose *τ* = 1/*α* = 1 at simulation steps Δ*t* = 0.0001; this corresponds to *τ*’ =10^4^ at Δ*t*′ = 1, and implies a very small distance to criticality *α*′ = 10^−4^). Thereby, the OU process introduces correlations among neurons and in time, and the resulting avalanche distributions display power laws with a cutoff. Overall, this choice of parameters makes the OU process more similar to our critical branching process than to a HPP [35].

Time varying external input may induce additional correlations not only for neural systems, but also in other collective systems, like the dynamics of flocks, which are subject to wind fluctuations and time varying external cues, or the dynamics of disease propagation that can be influenced seasonally, by weather conditions and by travel patterns. For all such systems, careful analyses are required to disentangle the external input from the internally generated dynamics. A classic example is that of solar flares, which evolve in cycles. Their inter event intervals (IEI) show a heavy tailed distribution. The generation of the heavy tail is derived from superposition of exponential distributions arising from different event rates [36,37], in analogy to the derivations here (Fig 3B).

**Fig 5:**
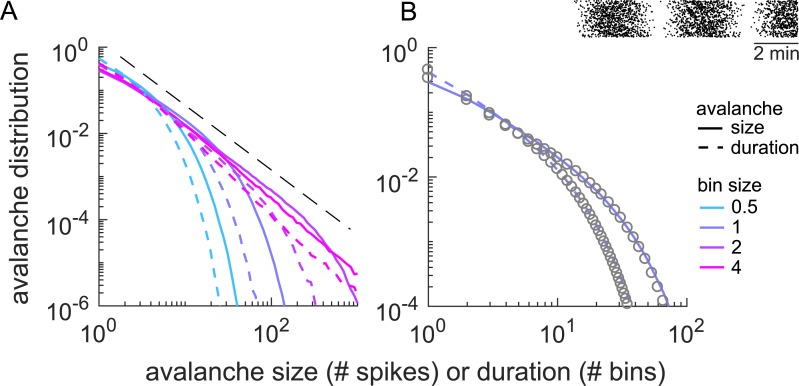
(Color) Avalanche size and duration distributions obtained for a continuously varying IPP are well approximated by power-law distributions with an exponent of -1.5 (A), and are well approximated by the analytical results, shown for bin size 1 (B). The IPP was realized as a sinusoidal with period T=250s and offset 1 (i.e. sin(t/T)+1), as sketched in the inset. The resulting mean rate is unity. Colored lines correspond to different bin sizes, circles depict analytical results, and the dashed black line depicts a reference power law with an exponent of -1.5.

For the generation of power laws from IPPs, we assumed that some external mechanism, the drive, makes the Poisson neurons fire with a fixed rate for a certain time interval, and then with a different rate for another time interval. For the simulations, the changes in *r* were assumed to be abrupt to allow for analytical treatment. However, the rate changes can also be slow and continuous. The important constraint is that the rates change slowly compared to the duration of an avalanche. In past studies, avalanches typically lasted a few milliseconds or tens of milliseconds (depending on the rate and bin size) [4,12,13,24,30]. Thus, any change in *r* of seconds can be considered “slow.” If the rate changed on very fast time scales, much shorter than typical avalanche durations, then the process would resemble an HPP with regard to the avalanche analysis. An example of a slowly varying drive is depicted in Fig 5, where we simulated a simple time-varying input, specifically, a sinusoidal with mean rate 1, amplitude 1, and a slow period of about four minutes. With this naïve choice of parameters, the avalanche size distribution approximated a power law with an exponent of -1.5 over three orders of magnitude (Fig 5A), and the numerical and analytical results still showed a good match (Fig 5B).

**Fig 6:**
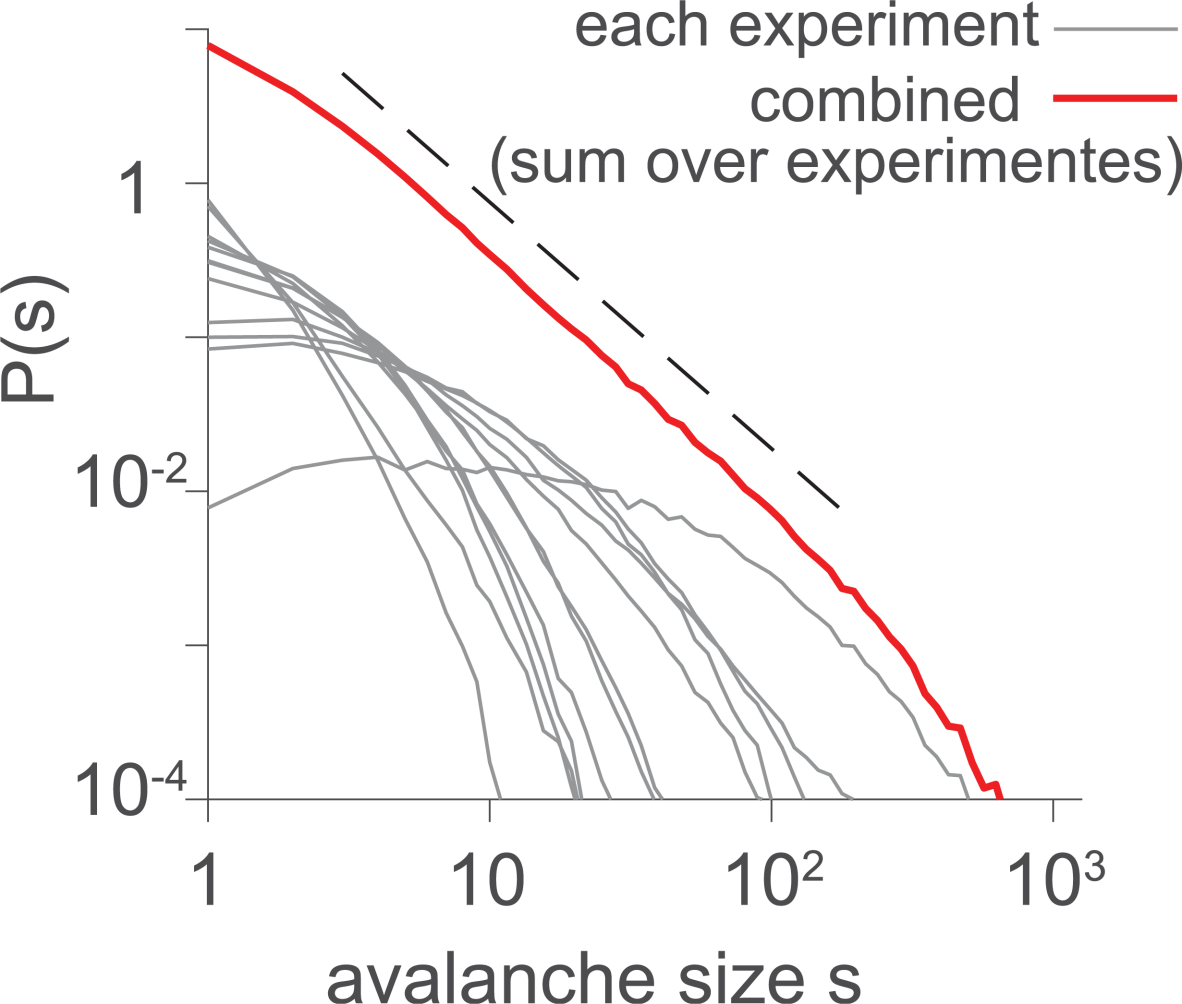
(Color) This graph illustrates that combining a number of size distributions recorded in different sessions or in different animals can easily yield approximate power laws. Depicted are the avalanche size distributions *P*_*S*_(*s*) from 12 spike recording sessions in Macaque monkeys used in [35] (gray; Δ*t* = 4 ms), and from summing over the 12 individual *P*_*S*_(*s*) (red; plotted with offset). Dashed line: power law with slope -1.6.

A power law could also arise from combining avalanche distributions from different experiments that differ in the mean event rate. Each recording might show an exponential distribution, but as the rates differ, the decay rates of the exponentials would differ, and adding them could yield approximate power law scaling. This effect is illustrated in Fig 6, where avalanche size distributions from 12 spike recording sessions in macaque monkeys were plotted both individually (gray) and in a combined manner (red). The data sets are precisely the same as those in [30,35]. The size distribution P(s) does not approximate a power law for any of the individual experiments, but combining the data from all twelve recording sessions yields a power law extending over more than two orders of magnitude. This is because each recording shows a different population spike rate, which translates to diverse decay behavior of P(s). Thus, it is evident that avalanche distributions from different experiments should not be combined into distributions by simple averaging. In contrast, an experiment in which the rate diversity lies in the Poisson neurons does *not* yield approximate power laws: If each neuron spikes with a different, constant Poisson rate, then the overall process is again an HPP with a firing rate equal to the sum of the individual rates.

Our current study of neural network dynamics using purely phenomenological models led us to ask: What can be achieved by using simple reduced models? We show here that such models offer an alternative explanation for power law generation: Instead of arising from critical networks, power laws can be imposed by a sophisticated drive with long time scales and large rate variations onto a set of unconnected Poisson neurons. Is this a better model for neural population dynamics? In terms of biophysical plausibility, certainly not: Single neuron dynamics are more complex than assumed here, and there is an abundance of connections between neurons and these connections are certainly used. Nonetheless, the phenomenological model allowed to disentangle the neural network dynamics generated within a network, and that imposed by external drive or input. A combination of the two determines the resulting population dynamics. Here we focused on the role of the external drive.

Long time scales have been observed in many studies (e.g., [2,4,38]). One argument for their emergence from within the network, and not from the external world, is that evidence for criticality has been found in isolated systems: *in vitro* networks clearly lack an external input but show evidence of internally generated criticality [4,10,11,24,39]. *In vivo* evidence for critical dynamics has also been provided for states with reduced input from the outside world, i.e., anesthesia and sleep in both animals and people [12,13,30]. In such a scenario, the long time scales could be imposed by input from a different part of the brain than the one recorded from, but these, in turn, need to generate the long time scales themselves. Thus, at least some brain areas need to generate the long time scales, e.g., by being close to criticality. In other words, the problem of generating long time scales is shifted only to a different entity than the one investigated, without solving the question about the origin of the long correlations. Importantly, the emergence of long time scales – indicative of near critical dynamics – has also been predicted in a detailed hierarchical model of the primate cortex [40].

A property of critical systems (with finite rate *r*) is the separation of timescales (STS). The STS imposes that the duration of an avalanche is typically much shorter than the pauses between avalanches. Assuming a certain rate *r*, a STS emerges in branching processes when approaching criticality. This is because the population rate *r* and the external input *h* obey the relation *r* = *h*/(1 − *σ*) = *h*/*ϵ*, where *ϵ* is the distance to the critical point. When approaching criticality (*ϵ* → 0), the drive rate *h* has to approach zero to assure a finite rate. Sufficiently close to criticality, the finite rate together with the diverging variance of the activity typically leads to long waiting times before a new avalanche is started and hence to a STS [35]. A STS implies that the avalanche size and duration do not change (much) when the bin size is changed, on condition that the bin size is shorter than the typical pauses (Fig 2E and 2F).

In experimental data, the waiting times or inter avalanche intervals (*IAI*), which are closely related to the inter event intervals (*IEI*) across all events, can reveal the nature of the external drive. For branching processes with Poisson drive, *p(IEI)* is exponentially distributed (Fig 3C). Different drives, however, would induce different *IEI* distributions. For the IPPs, for example, *p(IEI)* can resemble a power law (Fig 3B), or a Gamma distribution [36,37]. In experiments, both, approximate power laws [13,41,42], as well as exponential or gamma-like distributions [43,44] were observed. Thus the presence of a power-law distributed *p(IEI)* cannot prove a critical state, and the absence cannot rule out criticality. Similarly, temporal correlations between avalanche sizes have been observed in experiments and in some critical models, but not in all. Thus these correlations can narrow down the classes of generating models, but do not necessarily imply that the system is not critical.

Inference about the collective dynamics of a network in extended networks is further complicated if only a small fraction of all neurons can be sampled, or alternatively if one has to resort to coarse measures of neural activity such as LFP, EEG or MEG (coarse sampling) [35,39,43–45]. Currently, neural recordings *in vivo* are constrained by either subsampling or coarse sampling, and the biases that are potentially induced by sampling should be treated with care in any data analysis project. While no panacea exists to date to overcome these limitations, incorporating subsampling or coarse sampling to models, when comparing them to neural activity obtained from experiments is highly advisable. In fact, subsampling effects are already being implemented on a regular basis [13,14,30,35,43–46]. Recent advances have even provided an analytical understanding of subsampling-induced biases, which now allows us to correctly infer aggregated properties of a full system from an observed subset [35,39,47].

In conclusion, a non-critical system that is externally driven by a time-varying input can give rise to power law avalanche distributions resembling empirical distributions. The main requirements are that the rate envelope of the external drive changes sufficiently slowly in time, that it spans a wide enough range of rates, and that each rate contributes approximately for the correct fraction of time, given by *w(r)*. An important question concerns the general mechanisms that could give rise to such slowly varying temporal envelops. Ironically, one potential general mechanism is critical dynamics, which exhibits slow time scales. In other words, a system of non-interacting or weakly-interacting elements that are driven by a critical system may be indistinguishable from a genuine critical system. Thus, from the point of view of Occam's razor, it may well be that an underlying critical system is still the most parsimonious explanation of the data.

## Methods

### Ethics statement

The experiments were performed according to the German Law for the Protection of Experimental Animals and were approved by the Regierungspräsidium Darmstadt. The procedures also conformed to the regulations issued by the NIH and the Society for Neuroscience. The recordings were used in earlier publications already [30,35,48].

### Models

#### Homogeneous Poisson process

The spiking activity of our neural network model is simulated as a continuous-time, homogeneous (stationary) Poisson point process (HPP) with rate *r*. For avalanche analysis (see below), the process is transformed into discrete time steps t∈N by applying temporal bins Δ*t*. The number of events *A*(*t*) = *a* at each interval [*t*,*t* + Δ*t*) is then given by the Poisson distribution $P_{A}(a)=\frac{{\left(r\cdot \Delta t\right)}^{a}}{a!}e^{-r\cdot \Delta t}$. This process depends only on the product *r* ⋅ Δ*t*, and thus changes in *r* and changes in Δ*t* – while keeping the other parameter constant – have identical effects. Thus, throughout the manuscript, without loss of generality, we set either Δ*t* = 1 or *r* = 1 and vary only the other parameter. With Δ*t* = 1, the rate is given in units of 1/Δ*t*, and vice versa. In general, for any HPP, applying the same bin size *relative to the rate* yields exactly the same results.

For the standard avalanche analysis, which was introduced by Beggs & Plenz 2003 and is based on temporal binning, it is sufficient to generate just one random process *A*(*t*) to represent the activity of any *N* Poisson units, because the avalanche analysis does not require knowledge about the identity of the units (e.g., neuron, electrode, channel, or voxel): It combines the activity of all units into a single population activity vector *A*(*t*). To compare the Poisson process *A*(*t*) to neural activity, one can assume that Δ*t* = 1 ms and *r* = 1 kHz represents, for example, *N* = 100 independent Poisson neurons that each spike at rate 〈*r*_*i*_〉 = 10 Hz, or *N* = 256 EEG channels with an event rate on each channel of 〈*r*_*i*_〉 ≈ 3.9 Hz. Each of the units or channels can have a different rate; the only relevant parameter for Poisson activity is the rate *r* across all units: $r=\sum_{i=1}^{N}r_{i}=N\cdot \langle r_{i}\rangle$. We note that in addition to the conventional definition of avalanches using temporal binning, there are alternative definitions that assume spatial proximity and thus require knowledge about the identity of the units [49,50], or that make use of thresholding to separate one avalanche from another in the absence of clear pauses [51–53]. Here, we focus only on the classical temporal binning definition.

#### Inhomogeneous Poisson process

In many systems, such as the brain, it is conceivable that the event rate changes with time, i.e., *r* = *r*(*t*). For the analytical derivations, we assume that the rate changes slowly compared to the actual duration of the avalanches. In the example process that we use here, the rate *r*(*t*) assumed for a period of 250,000 time steps (≈ 4 min at Δ*t* = 1 ms) one of four different equiprobable rates, *r* = {1 2 5 10}/*Z*, where *Z* =18 assures that 〈*r*(*t*)〉 = 1 without loss of generality.

#### Critical branching process

In the context of criticality, activity propagation in the brain is commonly simulated using a branching model or branching process (BP) [4,30,31,35,39,43,45,54–56]. In a BP, each active unit *i* activates with some probability each of its postsynaptic units in the next time step. More precisely, each active unit *i* activates in the next time step *Y*_*t*,*i*_ = *y* units (called offsprings), where *Y* is a non-negative, integer random variable [constraints: *P*(*Y* = 0) > 0; *P*(*Y* = 0) + *P*(*Y* = 1) < 1]. Each of these activated units in the next time step again activates *y* units, leading to a cascade or avalanche-like propagation of activity. The dynamics of the process is defined by the control parameter *σ* = ∑_*y*_*P*(*y*) ⋅ *y* = 〈*Y*〉. For *σ* < 1 (> 1) the process is subcritical (supercritical), and for *σ* = 1 it is critical. Here, we realize the BP such that each active neuron activates with probability *q* = *σ*/*k* one of its *k* =2 postsynaptic neurons, i.e., *P*(*y*=0) = (1-*q*)^2^, *P*(*y*=1) = 2 *q*(1-*q*), *P*(*y*=2) = *q*^2^, and *P*(*y*>2) = 0. Note that all relevant measures in this paper are independent of the precise choice of the offspring distribution and depend only on *σ*. The number of events *A*(*t*) at each time step *t* is described as:

$$A(t)=\sum_{i=1}^{A(t-1)}Y_{i,t-1}+h_{t}$$ (17)

The external drive, *h*_*t*_, starts new “avalanches” with mean rate *h*. Here, we choose *h*_*t*_ to be 1 with probability *h* and zero otherwise. Given *h* > 0, the BP exhibits stationary dynamics in the subcritical regime (*σ* < 1), whereas in the supercritical regime it displays on average exponential growth, as expected. At criticality (*σ* = 1), it grows linearly. We choose a branching process with drive to approximate the dynamics of neural networks at criticality; this choice offers a number of advantages, apart from providing an elegant approximation of neural activity propagation: (a) It does not need to be mapped on a grid and thereby avoids finite size effects, (b) the distance *ε* to the critical point is well defined as *ε* = 1 − *σ*, and (c) the rate *r* of the stationary (subcritical) processes can be matched to that of empirical data or of other processes by adjusting the drive strength: *h* = *r* ⋅ *ε*.

In contrast to the Poisson processes, the BP is implemented on discrete time. To simulate a BP close to criticality but still in the stationary (subcritical) regime, we chose a fairly small distance to criticality *ε* = 1 − *σ* = 0.001. To make the BP comparable to the Poisson processes, we implement it with time steps of 0.25ms, and *r* = 1 kHz, where *r* = 〈*A*(*t*)〉. That rate is realized by using a drive *h* = *r* ⋅ *ε* = 0.001 per time step. Thus, on average, starting four new avalanches per second leads to an overall rate of 1 kHz. Recall, the units in (kHz) or (ms) are only for comparison to neural recordings and can be neglected.

### Experimental data—Spike recordings

The recording sessions are the same as in Priesemann et al. [30] and in Wilting & Priesemann [35]. The relevant details can be found in those articles and in the original publication of Pipa et al. [48]. In brief, spikes were recorded simultaneously from up to 16 single-ended micro-electrodes or tetrodes in the lateral prefrontal cortex of each of three trained macaque monkeys. For each recording, avalanches were extracted as described below, using a bin size of Δ*t* = 4 ms. In this study, we did not acquire new data but re-used data that had previously been recorded for different purposes. All relevant data are presented in this paper and in the Supporting Information files.

### Measures

Below we briefly review the definitions of avalanche measures and other time series measures. All definitions follow the standard definitions in the field. Most measures depend on the bin size Δ*t*, and hence Δ*t* introduces the relevant time scale for the time series.

**Fig 7:**
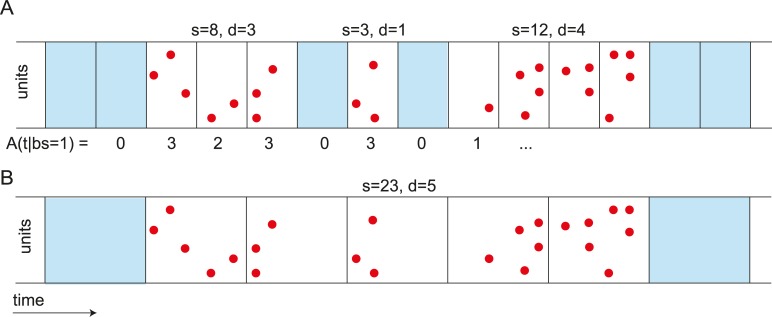
(Color). Avalanche Definition. **A**. For the avalanche analyses, events from all channels or units are combined into a single vector of activity per bin, *A(t|Δt)*, which is a function of the bin size (*Δt*). An avalanche is defined as the set of events in a sequence of non-empty time bins. Empty time bins are denoted in blue, events in red. The avalanche size *s* is defined as the total number of events in an avalanche, the avalanche duration *d* is defined as its length in bins (depicted above the raster plot). **B.** With changing the bin size, avalanche measures can also change (modified from [30]).

To define avalanches, events of all recorded units are combined into a single time series *A*(*t*), which describes the instantaneous population rate (Fig 7). To segment this time series into avalanches, temporal binning is applied. An avalanche is thus defined as a sequence of non-empty time bins, preceded and followed by at least one empty bin [4]. The avalanche size *s* is the total number of events in the avalanche, and the avalanche duration *d* is the number of non-empty bins in the sequence. Both quantities are expected to follow power law distributions with characteristic exponents if a system is critical [4,54]. The average avalanche size $\bar{s}$ given a duration *d* is denoted by $\bar{s}(d)$. The inter-event intervals (*IEI*) are defined as the time differences between subsequent events in the population rate vector *A*(*t*). The probability of observing *A* = *a* events in a time bin is denoted by *P*_*A*_(*a*|Δ*t*) or simply by *P*_*A*_(*a*). The Fano factor *F* is defined as the variance of the binned signal, divided by the mean. Both *A* and *F* depend on the bin size. Finally, the spike count ratio, *Q*, is defined as the ratio of events in the *i*^*th*^ bin, *A*(*t* = *i*|Δ*t*), and the previous bin, *A*(*t* = *i* − 1|Δ*t*), averaged over all bins with *A*(*t* = *i* − 1|Δ*t*) > 0:

$$Q=Q(\Delta t)=\langle \frac{A(i|\Delta t)}{A(i-1|\Delta t)}\rangle .$$ (18)

The measure *Q* is equivalent to the so-called “branching parameter” in Beggs & Plenz (2003) and subsequent studies; however, since the measure does not necessarily return the “branching parameter” of a branching process [25,30], we opted to give it a different name to avoid confusion.

### Analytical treatment

#### Derivation of avalanche size and duration distributions for a fixed-rate continuous time Poisson process

We assume that a sequence of independent discrete events is generated by a fixed-rate (homogeneous) Poisson process. The rate of the process is denoted by r.

A cascade or avalanche is defined as a sequence of consecutive time bins in which there was at least one event (Fig 7). The number of time bins in the sequence is the duration, denoted by *d*, and the total number of events is the size, denoted by *s*. Our goal is to calculate the size distribution, *P*_*S*_(*s*).

We first calculate the duration distribution, *P*_*D*_(*d*):

The probability of an empty bin is *p*_0_ = e^−rΔt^, and the probability of a non-empty bin is *p* = 1 − e^−rΔt^.

For simplicity and without loss of generality, we assume a time bin of one time unit, Δt = 1, which gives

$$p_{0}=e^{-r},\;p=1-e^{-r}$$

Due to the independence of different time bins, the probability of obtaining a sequence of d non-empty bins between two empty bins is proportional to $p^{d}p_{0}^{2}={\left(e^{-r}\right)}^{2}{\left(1-e^{-r}\right)}^{d}$. The normalization factor is the sum of a geometric series

$$Z_{D}={\left(e^{-r}\right)}^{2}\sum_{d=1}^{\infty }{\left(1-e^{-r}\right)}^{d}={\left(e^{-r}\right)}^{2}\frac{1-e^{-r}}{e^{-r}}={\left(e^{-r}\right)}^{2}(e^{r}-1)$$

Thus, the duration probability is given by

$$P_{D}(d)=\frac{1}{Z_{D}}{\left(e^{-r}\right)}^{2}{\left(1-e^{-r}\right)}^{d}=\frac{{\left(e^{r}-1\right)}^{d}}{e^{rd}(e^{r}-1)}=\frac{{\left(e^{r}-1\right)}^{d-1}}{e^{rd}}$$

The number of events in a single bin of a cascade, *A*, must be 1 or more. Thus, the distribution of the number of events in a single bin, Δt, is a renormalized Poisson distribution, which excludes the possibility of having 0 events:

$$P_{A\geq 1}(a)=\frac{1}{Z_{A\geq 1}}\frac{r^{a}e^{-r}}{a!}$$

where the normalization factor is given by

$$Z_{A\geq 1}=e^{-r}\sum_{a=1}^{\infty }\frac{r^{a}}{a!}=e^{-r}(e^{r}-1)=1-e^{-r}$$

In the last step, we have used the fact that the sum is the Taylor expansion of the exponential function excluding the first term. We thus obtain:

$$P_{A\geq 1}(a)=\frac{r^{a}}{(e^{r}-1)a!}$$ (19)

The total number of events in a cascade of duration *d* is the sum of *d* independent variables obeying the distribution *P*_*A*≥1_(*a*):

$$s=\sum_{i=1}^{d}a_{i}$$

The distribution of s given the duration *d* can be calculated from the corresponding generating function. The generating function of *P*_*A*≥1_(*a*) is given by:

$$G_{A\geq 1}(z)=\sum_{a=1}^{\infty }P_{A\geq 1}(a)z^{a}=\sum_{a=1}^{\infty }\frac{{\left(zr\right)}^{a}}{(e^{r}-1)a!}=\frac{e^{rz}-1}{e^{r}-1}$$

The generating function of a sum of independent random variables is the product of the underlying generating functions, yielding

$$G_{S}(z)=G_{A\geq 1}{\left(z\right)}^{d}=\frac{{\left(e^{rz}-1\right)}^{d}}{{\left(e^{r}-1\right)}^{d}}$$

We define

$$f(z)={\left(e^{rz}-1\right)}^{d}$$

Taking all this together and using the properties of a probability generating function, we obtain

$$P_{S}(s|d)=\frac{\partial^{s}f(z=0)/\partial z^{s}}{s!{\left(e^{r}-1\right)}^{d}}\equiv \frac{f^{\left(s\right)}(z=0)}{s!{\left(e^{r}-1\right)}^{d}}$$

To obtain the derivatives of *f*, we note that *f* is very similar in structure to

$$L(x)=\frac{{\left(e^{x}-1\right)}^{k}}{k!}=\sum_{n=k}^{\infty }\left\{\begin{array}{c} n\\ k \end{array}\right\}\frac{x^{n}}{n!}$$

the probability generating function of Stirling numbers of the second kind, $\left\{\begin{array}{c} n\\ k \end{array}\right\}$ [57]. These numbers describe the number of surjective ("onto") mappings of a set containing *n* elements onto a set containing *k* elements when *n* ≥ *k* (i.e., the number of mappings such that each of the *k* elements contains at least one of the *n* elements). They can be obtained from the generating function by

$$\left\{\begin{array}{c} n\\ k \end{array}\right\}=L^{\left(n\right)}(x=0)$$

Thus, the *s*^*th*^ order derivative of *f* at *z* = 0 is given by:

$$f^{\left(s\right)}(z=0)=u(d,s)r^{s}$$

where

$$u(d,s)=d!\left\{\begin{array}{c} s\\ d \end{array}\right\}=\sum_{i=0}^{d}{\left(-1\right)}^{i}(\begin{array}{c} d\\ i \end{array}){\left(d-i\right)}^{s}$$

This gives

$$P_{S}(s|d)=\frac{r^{s}d!\{\begin{array}{c} s\\ d \end{array}\}}{s!{\left(e^{r}-1\right)}^{d}}=\frac{r^{s}[\sum_{i=0}^{d}{\left(-1\right)}^{i}(\begin{array}{c} d\\ i \end{array}){\left(d-i\right)}^{s}]}{s!{\left(e^{r}-1\right)}^{d}}$$

The size distribution now can be expressed as

$$\begin{array}{l} P_{S}(s)=\sum_{d=1}^{s}P_{S}(s|d)P_{D}(d)\\ \;=\sum_{d=1}^{s}\frac{r^{s}[\sum_{i=0}^{d}{\left(-1\right)}^{i}(\begin{array}{c} d\\ i \end{array}){\left(d-i\right)}^{s}]{\left(e^{r}-1\right)}^{d-1}}{s!{\left(e^{r}-1\right)}^{d}e^{rd}}\\ \;=\frac{r^{s}}{s!}\frac{1}{\left(e^{r}-1\right)}\sum_{d=1}^{s}e^{-rd}\sum_{i=0}^{d}{\left(-1\right)}^{i}(\begin{array}{c} d\\ i \end{array}){\left(d-i\right)}^{s} \end{array}$$

We next calculate the mean avalanche size as a function of the duration, $\bar{s}(d)$. We first note that the mean number of events in a non-empty bin, *a*, satisfies

$$P(\mathrm{non-empty}\;\mathrm{bin})∙a+P(\mathrm{empty}\;\mathrm{bin})∙0=(1-e^{-r})∙a=r$$

Extracting *a* and multiplying by the duration, *d*, yields:

$$\bar{s}(d)=\frac{rd}{1-e^{-r}}$$

The mean avalanche size across all durations is given by:

$$\bar{s}=\sum_{d=1}^{\infty }P_{D}(d)\bar{s}(d)=\frac{r}{(1-e^{-r})e^{-r}}$$

The mean avalanche duration is given by:

$$\bar{d}=\sum_{d=1}^{\infty }P_{D}(d)d=\sum_{d=1}^{\infty }\frac{{\left(e^{r}-1\right)}^{d-1}}{e^{rd}}d=e^{r}$$

We note that for a power-law distribution with no cutoff and an exponent larger than -2, the mean avalanche size diverges. However, for a fixed-rate Poisson process, the distribution is not heavy tailed and the mean avalanche size is well defined.

The avalanche rate, i.e., the number of avalanches per time unit at a given rate is:

$$\rho \;(r)=\frac{r}{\bar{s}}=(1-e^{-r})e^{-r}$$

#### Exponential approximation

In general, the size distribution is non-monotonic. However, numerical simulations indicate that at large avalanche sizes the size distribution is approximately exponential, *P*_*S*_(*s*)~*e*^−*λs*^. We are interested in quantifying the dependence of the exponent on the rate of the underlying homogeneous Poisson process. Formally, the exponent can be estimated by evaluating

$$\lambda =\underset{s\to \infty }{\lim}\;-\log\;\frac{P_{S}(s+1)}{P_{S}(s)}=\underset{s\to \infty }{\lim}\;\left[\log\;P_{S}(s)-\log\;P_{S}(s+1)\right]$$

For a given *s*, the Stirling numbers obtain a single maximum value [57]. For a large *s*, the point at which the maximum is obtained and the maximum value itself can be approximated by

$$d^{*}\approx \;\frac{s}{\log\;s}$$

and

$$\log\;\left\{\begin{array}{c} s\\ d^{*} \end{array}\right\}\approx slog(s)-slog(\log\;s)-s$$

When summing over all values of d, the dominant contribution comes from d*, and the sum can be replaced by this dominant term. Using the above approximation and the Stirling approximation for factorial log *n*! ≈ *n*log(*n*) − *n*, we obtain:

$$\begin{array}{l} \log\;P_{S}(s)=\log\;\sum_{d=1}^{s}P_{S}(s|d)P_{D}(d)\approx \log\;P_{S}(s|d^{*})P_{D}(d^{*})=\log\;\left[\frac{r^{s}}{s!}\frac{d^{*}!e^{-rd^{*}}}{\left(e^{r}-1\right)}\{\begin{array}{c} s\\ d^{*} \end{array}\}\right]\\ \;=-\log\;\left(e^{r}-1\right)+s\;\log\;r-\frac{s}{\log\;s}(1+r)+\frac{s}{\log\;s}\log\;\frac{s}{\log\;s}-s\;\log\;\log\;s \end{array}$$

To estimate *λ*, we need to consider each term in the difference log *P*_*S*_(*s*) − log *P*_*S*_(*s* + 1) and evaluate its limit as *s* → ∞.

The first term vanishes in the difference, and for the second term the difference is −log *r*. For the third term, the limit of the difference is 0:

$$\underset{s\to \infty }{\lim}\;\left[\frac{s}{\log\;s}-\frac{s+1}{\log\left(s+1\right)}\right]=0$$

For the fourth term, the limit of the difference is -1:

$$\underset{s\to \infty }{\lim}\;\left[\frac{s}{\log\;s}\log\;\frac{s}{\log\;s}-\frac{s+1}{\log\;s+1}\log\;\frac{s+1}{\log\;s+1}\right]=-1$$

**Fig 8:**
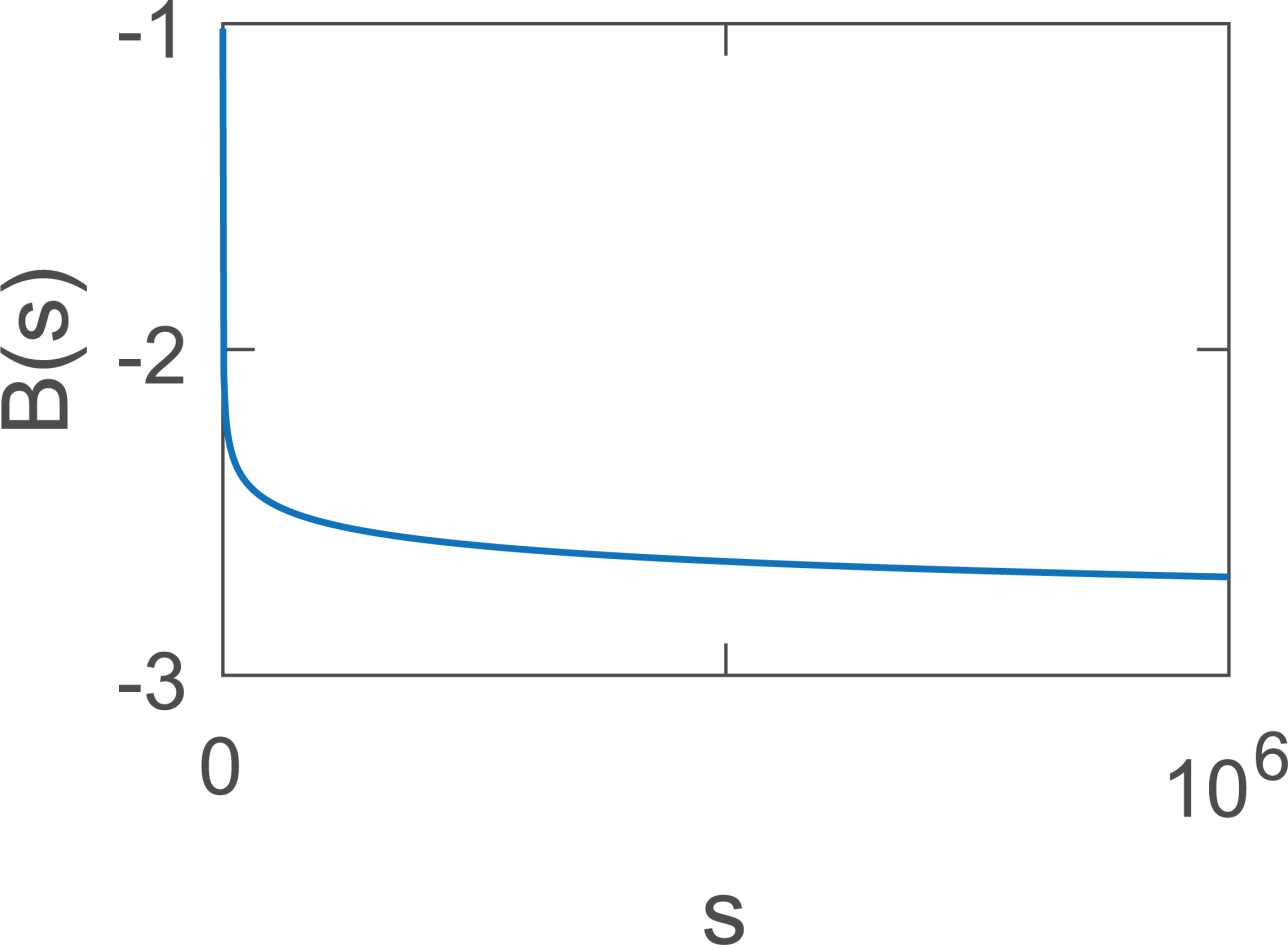
The function *B*(*s*) approaches its limit lim_*s*→∞_ *B*(*s*) = −∞ very slowly, and thus can be approximated by a constant for large intervals of *s*.

The last term changes very slowly with *s* due to the double log (Fig 8). Let us define it as:

B(s)=sloglogs−(s+1)loglog(s+1)

The limit of this term is −∞:

$$\underset{s\to \infty }{\lim}\;B(s)=-\infty$$

This relation shows that the size distribution is not strictly exponential but rather deviates slowly from a perfect exponential. Nevertheless, in practice one can replace *B*(*s*) → *B*(*s*_*c*_), where *s*_*c*_ is a representative value for the relevant range of sizes.

Taking all this together, we obtain the following approximation for the dependence of the exponent on the rate of the underlying Poisson process:

$$\lambda (r)=\underset{s\to \infty }{\lim}\;-\log\;\frac{P_{S}(s+1)}{P_{S}(s)}\approx -\log\;r-1+B(s_{c})$$

Thus, at large sizes *s*, the distribution can be approximated by:

$$P_{S}(s)\approx \lambda e^{-\lambda s}=(-\log\;r-1+B(s_{c}))\exp\;\left[-s(-\log\;r-1+B(s_{c}))\right]$$

To obtain a normalized distribution, *λ* must be positive. Thus, the following condition must be satisfied:

$$\lambda (r)=-\log\;r-1+B(s_{c})>0$$

logr<B−1

$$r<e^{B-1}$$

For sizes around 1000, *B* ≅ −2, giving *r* < 0.046. In other words, the exponential approximation is valid only for relatively small rates.

#### Avalanche size and duration distributions for an inhomogeneous Poisson process

For an inhomogeneous (time-dependent) Poisson process (IPP), the avalanche size distribution can be calculated by summing up the contributions from the different rates involved, assuming that the rate of the IPP changes slowly. To emphasize the dependence on the rate, we now explicitly denote the avalanche size distribution for a fixed-rate Poisson process (HPP) by *P*_*S*_(*s*|*r*). Given the temporal envelope of the rate, we denote the probability density function of the rates by *w*(*r*). The rate of avalanches during a Poisson process with a fixed-rate *r* is denoted by *ρ*(*r*) and derived above. The full avalanche size distribution of the IPP is given by

$$P_{S}(s)=\frac{\int_{0}^{\infty }dr\;w(r)\rho (r)P_{S}(s|r)}{\int_{0}^{\infty }dr\;w(r)\rho (r)}$$

This expression represents the portion of avalanches of size *s* out of the total number of avalanches of all sizes.

The duration distribution can be calculated in a similar way to the size distribution, giving:

$$P_{D}(d)=\frac{\int_{0}^{\infty }dr\;w(r)\rho (r)P_{D}(d|r)}{\int_{0}^{\infty }dr\;w(r)\rho (r)}$$

The dependence of the mean avalanche size on the duration is given by:

$$\bar{s}(d)=\frac{\int_{0}^{\infty }dr\;w(r)\rho (r)P_{D}(d|r)\bar{s}(d|r)}{P_{D}(d)\int_{0}^{\infty }dr\;w(r)\rho (r)}$$

The variance of the number of events *A* in a single bin is given by

$$\mathrm{var}(A)=\langle A^{2}\rangle -{\left\langle A\right\rangle }^{2}=\int_{r_{1}}^{r_{2}}dr\;w(r)(r+r^{2})-{\left(\int_{r_{1}}^{r_{2}}dr\;w(r)r\right)}^{2}$$

The Fano factor F of the number of events in a single bin is given by

$$F=\frac{\mathrm{var}(A)}{\mathrm{mean}(A)}=\frac{\int_{r_{1}}^{r_{2}}drw(r)(r+r^{2})-{\left(\int_{r_{1}}^{r_{2}}drw(r)r\right)}^{2}}{\int_{r_{1}}^{r_{2}}drw(r)r}$$

#### Derivation of the rate distribution that gives rise to a power law duration distribution

As shown above, for an HPP, the duration distribution is an exponential distribution, and the corresponding size distribution is approximately exponential. An IPP can give rise to a power law distribution if the distribution of its underlying rates, *w*(*r*), has a specific form. Below we derive the rate distribution that gives rise to a power-law duration distribution.

Using the expressions for *ρ*(*r*) and *P*_*D*_(*d*|*r*), we obtain:

$$P_{D}(d)∼\int_{0}^{\infty }dr\;w(r)\rho (r)P_{D}(d|r)=\int_{0}^{\infty }dr\;w(r)(1-e^{-r})e^{-r}\frac{{e^{-r}(1-e^{-r})}^{d}}{1-e^{-r}}=\int_{0}^{\infty }dr\;w(r)e^{-2r}{\left(1-e^{-r}\right)}^{d}$$

We next define

$$\mu =-\log\left(1-e^{-r}\right)$$

Changing the integration variable from *r* to *μ*, we obtain

$$dr=\frac{1-e^{-r}}{e^{-r}}d\mu ,\;\mu (r=0)=\infty ,\;\mu (r=\infty )=0$$

$$P_{D}(d)∼\int_{0}^{\infty }d\mu \;w(r(\mu ))(1-e^{-r(\mu )})e^{-r(\mu )}e^{-\mu d}$$ (20)

We next show that a superposition of exponential distributions with a power law weighting function can yield a power law distribution. This distribution can be derived from the properties of the gamma function. The lower incomplete gamma function satisfies the following relationship:

$$\int_{t_{1}}^{t_{2}}dt\;t^{\beta -1}e^{-t}=\gamma (\beta ,t_{2})-\gamma (\beta ,t_{1})$$

where the lower incomplete gamma function is defined as:

$$\gamma (\beta ,x)=\int_{0}^{x}dt\;t^{\beta -1}e^{-t}$$

Changing the integration variable to *μ* = *t*/*d*, we obtain:

$$\int_{\mu_{1}}^{\mu_{2}}d\mu \;\mu^{\beta -1}e^{-\mu d}=d^{-\beta }[\gamma (\beta ,d\mu_{2})-\gamma (\beta ,d\mu_{2})]$$ (21)

Comparing Eqs (20) and (21), we obtain the following expression for the weighting function:

$$w(r)=\frac{{\left[-\log\;\left(1-e^{-r}\right)\right]}^{\beta -1}}{{(1-e^{-r})e}^{-r}}$$

The resulting duration distribution is then given by:

$$P_{D}(d)∼d^{-\beta }[\gamma (\beta ,-d\;\log\;\left(1-e^{-r_{1}}\right))-\gamma (\beta ,-d\;\log\left(1-e^{-r_{2}}\right))]$$

where *r*_1_ and *r*_2_ are the lower and upper bounds of the rate distribution, respectively. Note that the lower rate,*r*_1_, is associated with the upper *μ* value and vice versa. Taking the limit *r*_1_ → 0, *r*_2_ → ∞, would yield a perfect power law. However, in this limit *w*(*r*) cannot be normalized. Moreover, in practice, there would be some lower and upper bounds to the rate distribution, and hence the duration distribution would deviate from a perfect power law.

For critical branching processes, the exponent of the duration distribution is *β* = 2, which can be obtained by using

$$w(r)=\frac{-\log\left(1-e^{-r}\right)}{{(1-e^{-r})e}^{-r}}$$

#### Derivation of the spike count ratio Q

We here derive the dependence of the spike count ratio Q on the rate *r* of a Poisson process (which is equivalent to changes in the bin size). Assuming Δ*t* = 1, the general definition of Q is

$$Q(r)=\sum_{k=0}^{\infty }\sum_{l=1}^{\infty }\frac{k}{l}P_{k,l}(k|l)P_{l}(l),$$

where *P*_*k*,*l*_(*k*|*l*) is the probability of having k events in a bin and *l* events in the previous bin. Note that the sum over *l* starts only at 1, by definition of *Q*.

For Poisson processes, the probabilities for obtaining *A* = *k* or *A* = *l* events in a time bin is independent of the number in the previous bin. Therefore, for Poisson processes *Q*_*P*_(*r*) becomes:

$$Q_{P}(r)=\sum_{k=0}^{\infty }\sum_{l=1}^{\infty }\frac{k}{l}P_{A\geq 0}(k)P_{A\geq 1}(l)$$

where *P*_*A*≥0_(*k*) is the regular Poisson distribution and *P*_*A*≥1_(*l*) is the renormalized Poisson distribution, which appears in Eq (19). Inserting these relations and executing the sums, we obtain:

$$\begin{array}{l} Q_{P}(r)=\sum_{k=0}^{\infty }\sum_{l=1}^{\infty }\frac{k}{l}\frac{1}{Z_{A\geq 0}}\frac{r^{k}e^{-r}}{k!}\frac{1}{Z_{A\geq 1}}\frac{r^{l}e^{-r}}{l!}\\ =\sum_{k=0}^{\infty }\sum_{l=1}^{\infty }\frac{k}{l}\frac{r^{k}e^{-r}}{k!}\frac{1}{1-e^{-r}}\frac{r^{l}e^{-r}}{l!}\\ =\frac{r(\ln\;\left(r\right)+\gamma -Ei(r))}{1-e^{r}} \end{array}$$

*γ* is the Euler-Mascheroni constant (*γ* ≈ 0.577) and $Ei(r)=-\int_{-r}^{\infty }\frac{e^{-t}}{t}dt$ is the exponential integral function.

Note that here, as usual, *r* can be exchanged with Δ*t* assuming Δ*t* = 1 or *r* = 1, respectively. Thus *Q*_*P*_(*r*) = *Q*_*P*_(*r*|Δ*t* = 1) = *Q*_*P*_(Δ*t*|*r* = 1). More generally,

$$Q_{P}(\Delta t,r)=\frac{r\cdot \Delta t\;(\ln\left(r\cdot \Delta t\right)+\gamma -Ei(r\cdot \Delta t))}{1-e^{r\cdot \Delta t}}$$

## Supporting information

S1 Data Spike avalanche distributions of Fig 6. Spike avalanche distributions from recordings in prefrontal cortex of macaque monkey. Details on the recordings can be found in Priesemann et al., 2014 [30].
(ZIP)
